# Salinity Tolerance in Freshwater Drum (*Aplodinotus grunniens*): Investigating Biochemical, Antioxidant, Digestive Enzyme, and Gene Expression Responses to Acute Salinity Stress

**DOI:** 10.3390/ani15071015

**Published:** 2025-04-01

**Authors:** Justice Frimpong Amankwah, Wu Jin, Xueyan Ma, Pao Xu, Haibo Wen, Kennedy Emeka Amuneke, Brian Pelekelo Munganga, Kang Li, Jingwei Liu, Hongxia Li

**Affiliations:** 1 Wuxi Fisheries College, Nanjing Agricultural University, Wuxi 214081, China; jinw@ffrc.cn (W.J.); maxy@ffrc.cn (X.M.); xup@ffrc.cn (P.X.); lihx@ffrc.cn (H.L.); 2 Key Laboratory of Integrated Rice-Fish Farming Ecology, Ministry of Agriculture and Rural Affairs, Freshwater Fisheries Research Center, Chinese Academy of Fishery Sciences, Wuxi 214081, China; 3 Sino-US Cooperative Laboratory for Germplasm Conservation and Utilization of Freshwater Mollusks, Freshwater Fisheries Research Center, Chinese Academy of Fishery Sciences, Wuxi 214081, China; 4 China-ASEAN “The Belt and Road” Joint Laboratory of Marine Culture Technology (Shanghai), Shanghai Ocean University, Shanghai 201306, China; kli@shou.edu.cn (K.L.); kivi.liu@outlook.com (J.L.); 5 Key Laboratory of Exploration and Utilization of Aquatic Genetic Resources, Ministry of Education, Shanghai Ocean University, Shanghai 201306, China; ke.amuneke@unizik.edu.ng; 6 Department of Fisheries and Aquaculture, Nnamdi Azikiwe University, Awka 422001, Nigeria; 7 Center for Innovative Approach Center Zambia, Lusaka 10101, Zambia; brijelimunga@gmail.com; 8 Center for Ecological Aquaculture (CEA), Shanghai Ocean University, Shanghai 201306, China; 9 Shanghai Engineering Research Center of Aquaculture, Shanghai Ocean University, Shanghai 201306, China

**Keywords:** *A. grunniens*, salinity tolerance, physiological response, brackish water adaptation

## Abstract

Variations in salinity levels in aquaculture significantly influence fish physiology, impacting population dynamics and industry viability. This study aimed to examine the physiological response of the freshwater drum (*Aplodinotus grunniens*) to differing salinity conditions, assessing its potential for cultivation in brackish water environments. Fish averaging 45 ± 0.1 g were subjected to acute salinity tests across three groups: a control group at 0‰ and experimental groups at 7.5‰ and 15‰ over four days. The initial findings indicated that *A. grunniens* could tolerate salinity levels up to 15‰ without adverse effects. Key biochemical markers, such as aspartate aminotransferase and alanine aminotransferase, exhibited significant fluctuations but decreased over time. Antioxidant enzyme activity increased relative to the control, while malondialdehyde levels declined, indicating effective oxidative stress management. Additionally, digestive enzymes like amylase and lipase demonstrated adaptability to changing salinity. The expression of heat shock proteins 70 and 90 in the gills and livers varied initially but showed no sustained changes. Overall, the results suggest that *A. grunniens* possesses notable resilience to salinity variations, indicating its suitability for brackish water aquaculture and highlighting the optimal salinity ranges for promoting growth.

## Simple Summary

This study explored the physiological response of freshwater drum (*Aplodinotus grunniens*) to acute changes in salinity levels (0‰, 7.5‰, and 15‰) over four days, aiming to evaluate its potential for aquaculture in brackish water environments. Initial biochemical changes, including elevated levels of aspartate aminotransferase, alanine aminotransferase, superoxide dismutase, catalase, and sodium–potassium adenosine triphosphate, were observed but largely stabilized over time, indicating an ability to adapt. Levels of malondialdehyde decreased, suggesting the effective management of oxidative stress. Digestive enzyme activity adjusted to the different salinity conditions. Although the expression of heat shock proteins 70 and 90 varied initially, no sustained changes were noted. These findings indicate that *Aplodinotus grunniens* exhibits substantial tolerance to salinity, making it a promising candidate for brackish water aquaculture. However, further studies are required to determine the optimal salinity levels for maximizing growth and health.

## 1. Introduction

Aquaculture, the practice of farming aquatic organisms such as fish, shellfish, and plants, is rapidly expanding globally, with fish farms now contributing to roughly half of the world’s fish production [1]; however, a major obstacle faced by this industry is the issue of managing water salinity levels and the growing scarcity of freshwater, particularly in regions where freshwater sources are limited for large-scale fish farming operations [2]. This situation is due to water scarcity and climate change, which includes rising temperatures that directly increase water evaporation and lead to higher salinity levels combined with human activities like industrial and agricultural waste in freshwater ecosystems [3]. Furthermore, changes in salinity significantly affect the physiological functioning of fish, reflecting a key environmental factor contributing to the decline of the fish industry. Moreover, these fluctuations in salinity primarily cause harmful alterations in fish hormones and enzymes, impacting their overall health and survival ability [4].

When salinity in water deviates significantly from a fish’s optimal range, it causes “osmotic stress”, disrupting their normal bodily functions and forcing them to expend extra energy to maintain their internal salt balance, ultimately impacting their growth, physiology, feeding behavior, appetite, digestive enzyme activity, and survival, impacting overall health and productivity regardless of whether the salinity is too low or too high [5,6,7,8]. While salinity plays a crucial role in ecosystems and can be significantly impacted by climate change, scientists and researchers often focus more on factors like temperature and carbonate chemistry when discussing the effects of global environmental shifts, potentially overlooking the critical influence of salinity variations [9]. For example, changes in salinity can disrupt the natural balance of an ecosystem and cause physiological stress in carp, which disrupts their internal bodily functions and potentially causes large-scale deaths due to a sudden change in osmotic pressure [10]. When an organism is exposed to increased salinity in its environment, it maintains internal balance (homeostasis) by actively pumping more sodium (Na+) and potassium (K+) ions into its bloodstream, primarily through the increased activity of sodium–potassium adenosine triphosphatase (Na+/K+-ATPase) enzyme located in the gills, which actively transports these ions across cell membranes [11]. Research has investigated the role of Na+/K+-ATPase in various fish species, including the gills and kidneys of silver catfish, Nile tilapia, Japanese eel, Senegalese sole, and pufferfish, as demonstrated by multiple studies [12,13,14,15]. Sudden changes in salinity levels can induce oxidative stress, characterized by the excessive production of harmful reactive oxygen species (ROS). Initially, this overwhelms the antioxidant defense system, impairing the fish’s ability to counteract the damaging effects of ROS and increasing susceptibility to infections [16,17].

In response, fish activate physiological mechanisms to restore homeostasis by enhancing the production of antioxidant enzymes, including superoxide dismutase (SOD), catalase (CAT), and total antioxidant capacity (T-AOC). They help maintain homeostasis in fish by neutralizing ROS when the fish encounter different stressors, such as fluctuations in temperature and salinity [18,19,20,21]. Cells respond to stress by producing cytoprotective proteins, known as heat shock proteins (HSPs). HSPs are released into the bloodstream and fish tissues in response to environmental changes [22,23]. According to recent research, heat stress is not the only stressor that causes HSPs to become more sensitive in all cells [24,25,26,27,28]. Toxins, anoxia, hypoxia, and salinity can also cause this sensitivity increase [29,30,31]

The study hypothesized that optimal salinity levels can significantly improve the physiology and health of organisms. Specifically, acute exposure to varying salinity levels is expected to positively influence the well-being of the species under study. This will essentially bridge the knowledge gap that freshwater drum cannot survive in hypersalinity exposure. Ultimately, this study aims to understand how well *A. grunniens*, the only species in its family to live in freshwater conditions permanently, can adapt to different salinity levels, particularly in extreme environmental changes like freshwater salinization, pollution, and water scarcity, by investigating the underlying physiological mechanisms to help restore disturbed ecosystems impacted by salinity fluctuations. Therefore, evaluating parameters such as biochemical enzyme levels, digestive enzymes, and changes in gene expressions in fish are considered appropriate markers to assess the potential impact of increased salinity exposure on their antioxidant tolerance and molecular mechanisms; this is because changes in these parameters can indicate stress and compromise defense mechanisms when fish are exposed to salinity levels beyond which their body can accommodate [32].

## 2. Materials and Methods

### 2.1. Ethics Statement

This research was approved by the Ethics Committee of Nanjing Agricultural University, Nanjing, China, and Freshwater Fisheries Research Center (FFRC), CAFS (NO. 2020JBFR02, 2020JBFR03, and 2021JBFM13)

### 2.2. Fish Husbandry and Experimental System

*A. grunniens* were sourced from the ponds of “Jiangyin” (120.124132° E, 31.91298° N), an experimental base of Wuxi Fisheries College of Nanjing Agricultural University (NAU), and transferred to FFRC. All the experimentation and fish husbandry were performed accordingly in compliance with the research ethical guidelines of NAU.

To commence the experimental trials, nine distinct salinity concentrations (0, 5, 7.5, 10, 12.5, 15, 17.5, 20, and 25‰) were prepared, and for each concentration, 10 fish were stocked and observed for five days. These initial experiments were conducted without feeding the fish, with the primary objective being to determine the salinity concentration at which mortality would occur. Each tank utilized for this purpose had a water capacity of 140 L with three replicates. This 120 h exposure experiment was conducted following American Public Health Association (APHA) standard procedures [33] utilizing the direct transfer method [34], and observed for 5 days.. At regular intervals, multiple checks were conducted, and dead fish were promptly removed upon discovery. The fish were declared dead when there was a cessation of opercular respiratory movement and no response to touch stimulation.

**Figure 1:**
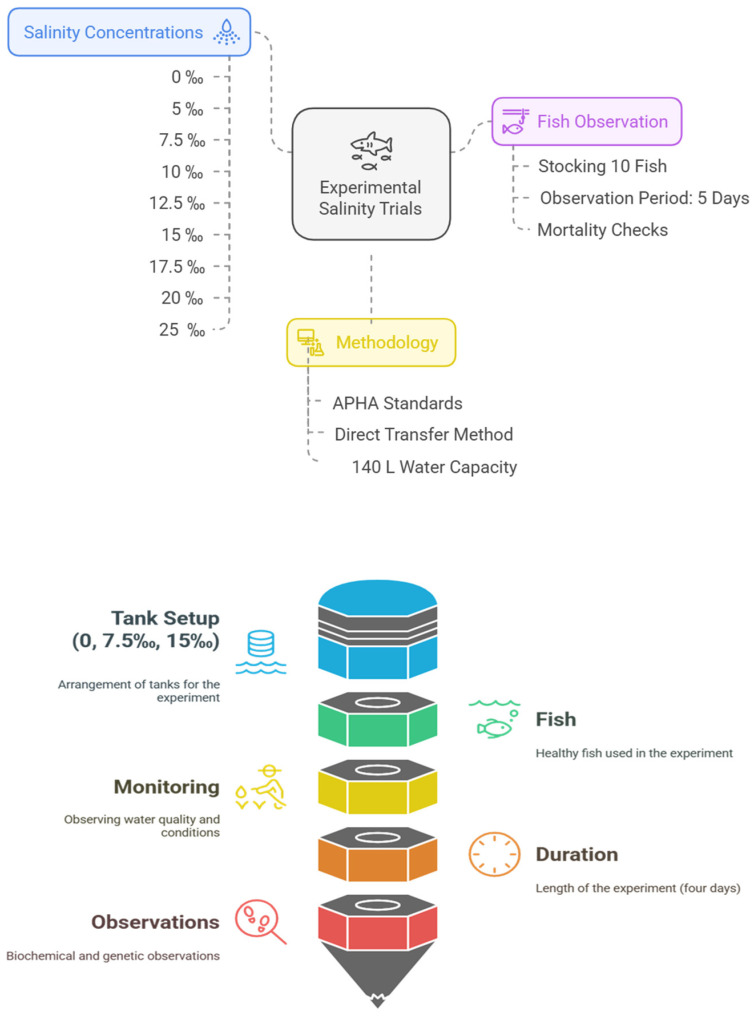
Experimental design.

An acute salinity experiment was then set using three indoor tanks, each containing 15 fish of similar size (45 ± 0.1 g), and a total water capacity of 140 L randomly assigned to healthy fish. These tanks represented varying salinity levels: a control tank with 0‰ salinity and two experimental tanks with salinities of 7.5‰ and 15‰, respectively. This acute exposure experiment included three replicate tanks for each treatment group and lasted four days without feeding the fish. Throughout the experiment, water quality parameters and photoperiod conditions were continuously monitored. The desired salinity levels in all 3 experiments were established by dissolving a measured quantity of Haibo reef salt purchased from Jiangxi Yantong Technology in a designated volume of inland freshwater, and their accuracy was subsequently verified using a salinometer. Adjustments were made as necessary to achieve the desired salinity levels. Checks were performed on dissolved oxygen and pH levels, while the temperature was monitored daily (Temperature 22 °C, the dissolved oxygen (DO) at 6.1 ± 0.5 mg/L, and the pH at 7.1, 12 h:12 h light/dark cycle was maintained). Ammonia-nitrogen and nitrate-nitrogen concentrations were measured using a HACH Spectrophotometer, while the standard level of alkalinity and hardness of the saline water were ensured within the accepted recommendation of APHA [33]. Figure 1 displays the whole experiment design.

### 2.3. Sample Collection

The fish were anesthetized using MS-222 at a concentration of 200 mg/L. Following this, the fish underwent dissection, during which blood samples, gills, livers, and intestinal samples were collected and weighed to determine the biochemical markers, digestive enzyme activities, and gene expressions, respectively. Three replicates of the sample were collected at each of the following sampling times: 0, 4, 12, 24, 48, and 96 h. Immediately after collection, all the samples were carefully sorted and promptly immersed in liquid nitrogen. They were then transferred to a cryogenic freezer set at −80 °C for storage until further analysis.

### 2.4. Liver Function and Antioxidant Enzyme Activities

With sharp scissors, blood samples were obtained from the ventral area of the fish, and secured in position with a damp cloth. Subsequently, a portion of the blood was centrifuged at 3000 rpm for 4 min to separate the supernatant fraction for hematological analysis. The levels of aspartate aminotransferase (AST) and alanine aminotransferase (ALT), along with the biochemical markers related to the antioxidant status, were assessed following the guidelines provided by the Nanjing Jiancheng Bioengineering Institute, Nanjing, China. The protocol described by Jiang et al. [35] was employed to determine the activity of the enzyme CAT. The quantification of malondialdehyde (MDA) concentration was carried out using the method outlined by Zhang et al. [36]. Additionally, the measurement of SOD activity was performed according to the procedure detailed by Wang and Chen [37]. The T-AOC was assessed following the methods outlined by Marklund and Marklund [38]. The activity of the Na+/K+-ATPase enzyme was evaluated to detect the level of inorganic phosphorus generated. Specifically, the conversion of ATP to ADP and inorganic phosphate is catalyzed by the Na+/K+-ATPase enzyme. Inorganic phosphate released over time serves as a proxy for measuring the enzymatic activity of Na+/K+-ATPase. After the enzyme reaction, freed inorganic phosphorus is processed, and the activity rate is determined by spectrophotometrically measuring the absorbance at 636 nm. This linked enzymatic assay enables the indirect measurement of Na+/K+-ATPase activity by identifying the byproduct of inorganic phosphate produced when the enzyme hydrolyzes ATP in the assay.

### 2.5. Digestive Enzyme Activity Assay

The gut samples were processed following the protocol outlined by Gisbert et al. [39]. Before analysis, all the tissue samples were precisely weighed. Each sample was then mixed with 0.9% precooled physiological saline at a 1:10 weight–volume ratio (grams to milliliters) [39]. This mixture was homogenized using a high-speed Kinematica Model MB 550 grinder. The resulting homogenate was subsequently centrifuged at 4 °C for 10 min at 2500 rpm. The supernatants obtained from the intestinal and stomach tissue homogenates were utilized for measuring digestive enzyme activity [39]. The following criteria were established to define one unit of enzyme activity:

- Amylase: The quantity of enzyme capable of hydrolyzing 10 mg of starch within 30 min at 37 °C in the presence of 1 mg of tissue protein [40].
- Lipase: The amount of enzyme that can process 1 µmol of substrate per minute in a reaction containing 1 g of tissue protein at 37 °C [41].
- Pepsin: The enzyme quantity that can break down protein to yield an equivalent of 1 µg of amino acids per minute per 1 mg of tissue protein at 37 °C [41].

### 2.6. Extraction of RNA and Purification Measurement

The liver and gill tissues from *A. grunniens* were utilized for total RNA extraction. Tissue samples weighing 50 to 100 mg were placed in sterile 2 mL tubes, followed by adding 1 mL of Trizol pure isolation reagent. The homogenization of the samples was achieved using steel balls at a frequency of 50 Hz for 45 s. After homogenization, the tubes were allowed to cool for 5 min at room temperature. Subsequently, 200 µL of chloroform was added to each homogenized tissue sample, and the mixture was left to settle for 5 min before centrifugation at 12,000× *g* for 15 min at 4 °C. After centrifugation, the RNA-containing supernatant was carefully transferred to new tubes. Isopropanol (500 μL) was added to precipitate the RNA, left to settle for 10 min, then centrifuged at 12,000 g for 15 min at 4 °C. The resulting supernatant was discarded, and the RNA pellet was washed with 1 mL of 75% ethyl alcohol. Following washing, the samples were centrifuged at 7500× *g* for 5 min at 4 °C, and the supernatants were removed. The RNA pellets were air-dried for 20–30 min. Subsequently, the extracted RNA was dissolved in 30 μL of Dnase-/RNase-free water and stored at −80 °C for further use.

Quality control assessment was conducted using a Nanodrop-NV3000 spectrophotometer (Thermo Scientific, Waltham, MA, USA). This involved analyzing absorbance ratios at 260/280 nm and 260/230 nm to ensure RNA purity and quality, with the ratios typically falling between 1.7 and 2.1, indicating minimal contamination from proteins, phenolic compounds, or other impurities. Additionally, the RNA sample concentrations were determined using the same spectrophotometric analysis. This step was to ensure that sufficient quantities of high-quality, homogeneous RNA were available for the subsequent synthesis of complementary deoxyribonucleic acid (cDNA).

### 2.7. cDNA Synthesis, Primer Sequences, and Real-Time PCR Analysis (qRT-PCR)

Following RNA isolation, quantitative reverse transcription PCR was conducted using a PrimeScriptTM RT reagent kit (Takara, Dalian, China) for cDNA synthesis, adhering to the manufacturer’s instructions. β-actin was chosen as the reference gene due to its stable expression. A 20 μL reaction volume was prepared by combining 2.0 μL of cDNA, 10.0 μL of SYBR^®^ Premix Ex Taq II (2×), 0.8 μL of forward and reverse primers (10 μM each), 0.4 μL of ROX reference dye II (50×), and 6.0 μL of RNase-free distilled water. Table 1 provides the specific primer sequences for the genes under investigation following the methods outlined by Pfaffl [42].

### 2.8. Statistical Analysis

Statistical analyses were performed using GraphPad Prism version 8.0. Prior to the analyses, data were tested for normality using the Shapiro–Wilk test and for homogeneity of variance using Levene’s test. A two-way analysis of variance (ANOVA) was conducted to assess the effects of different salinity levels on biochemical and digestive enzyme activities, as well as gene expression responses in *A. grunniens*. When significant effects were observed, Tukey’s post hoc test was applied to determine pairwise differences between the treatments. The results are presented as mean ± standard error (SE), and statistical significance is set at *p* < 0.05.

## 3. Results

### 3.1. Observation of Mortalities

**Figure 2:**
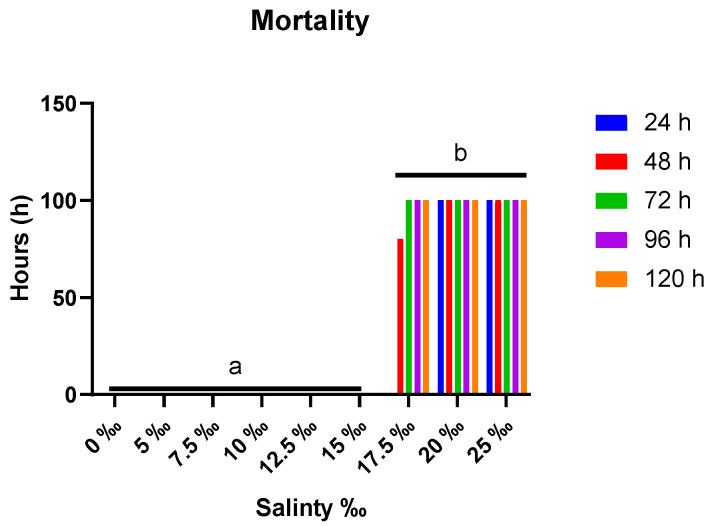
Mean mortality in *A. grunniens* under varying salinity conditions across different time points. Data are presented as means with different lowercase letters indicating statistically significant differences between salinity treatments (*p* < 0.05), while matching letter(s) indicate no significant difference according to two-way ANOVA. No mortality was observed at salinity levels of 0‰ to 15‰, while 100% mortality was recorded above 15‰ after 48 h.

Notable observations from the initial experiment in Figure 2 revealed the different levels of mortalities after the species were subjected to nine different salinity levels. *A. grunniens* exhibited normal swimming behavior and survived at salinity levels up to 15‰. However, mortality rates escalated proportionally beyond this threshold. When the fish were exposed to salinities higher than 15‰, many aberrant behaviors were noted in them. These included hyperactivity, seizures, increased opercular movement, and swimming toward the water’s surface, among other stress-related behaviors in fish [43,44]. To summarize, eighty percent (80%) mortalities were observed after 24 h, and a one hundred percent mortality (100% mortality) after 48 h in the 17.5‰ group. Beyond the 17.5‰ salinity level, one hundred percent (100%) mortalities were observed after 24 h.

### 3.2. Liver Function Enzyme Activities

**Figure 3:**
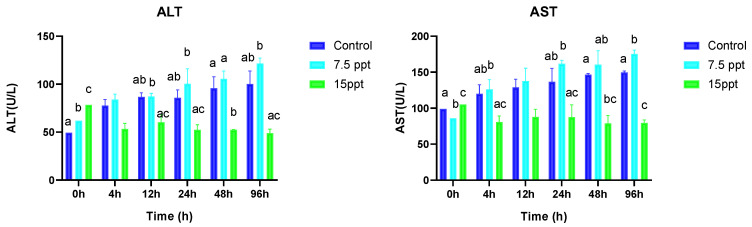
Mean activities of alanine transaminase (ALT) and aspartate transaminase (AST) in *A. grunniens* under varying salinity conditions across different time points. Data are presented as means and standard errors (SEs). Different lowercase letters indicate statistically significant differences between salinity treatments (*p* < 0.05), while matching letters indicate no significant difference according to two-way ANOVA. A combination of letters (e.g., ‘ab’) indicates that the group shares statistical similarities with both the ‘a’ and ‘b’ groups.

The salinity levels and sampling times significantly influenced the ALT and AST activities (Figure 3). Substantial differences in the ALT activity were observed at 0 h between the treatment groups and the control (*p* < 0.05). At the subsequent sampling times, significant differences were found only between the control and 15‰ group, notably at 48 h (*p* < 0.05). The AST activity showed considerable variation at 0 h across the treatments (*p* < 0.05). From 4 to 96 h, significant differences occurred between the control and both the 7.5 and 15‰ groups, with the highest difference in the 7.5‰ group and the lowest in the 15‰ group at 96 h (*p* < 0.05).

### 3.3. Antioxidant Enzyme Activities and Na+/K+-ATPase

**Figure 4:**
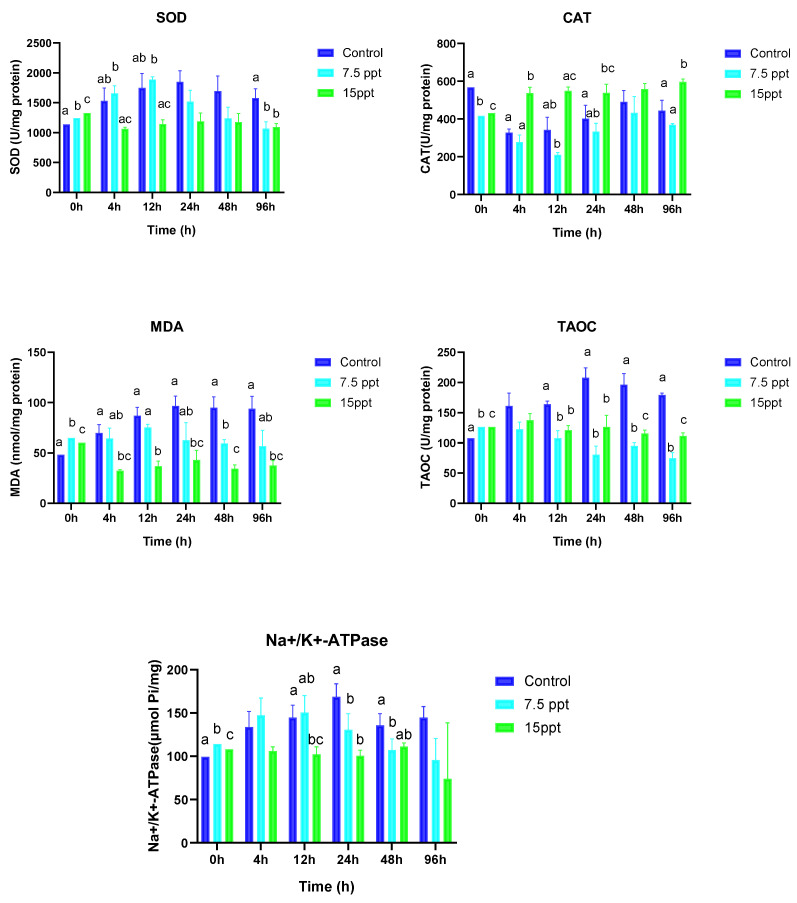
Mean activities of superoxide dismutase (SOD), catalase (CAT), malondialdehyde (MDA), total antioxidant capacity (TAOC), and sodium–potassium adenosine triphosphate Na+/K+-ATPase in *A. grunniens* under varying salinity conditions across different time points. Data are presented as means and standard errors (SEs). Different lowercase letters indicate statistically significant differences between the salinity treatments (*p* < 0.05), while matching letters indicate no significant difference according to two-way ANOVA. A combination of letters (e.g., ‘ab’) indicates that the group shares statistical similarities with both the ‘a’ and ‘b’ groups.

Figure 4 provides a comprehensive overview of how salt and sampling time influence the antioxidant activities of SOD, CAT, MDA, TAOC, and Na+/K+-ATPase in *A. grunniens*. At the beginning of sampling (0 h), significant differences were observed in the treatment groups and the control for all the antioxidant activities (*p* < 0.05). From the 4 h sampling time, SOD activities displayed no significant changes except at the 96 h sampling time, where significant changes were observed between the control and 7.5‰ group as well as the control and 15‰ group (*p* < 0.05). At the 4 h sampling time, significantly higher differences were observed in the 15‰ group compared to the control (*p* < 0.05), whereas no differences were observed between the control and the 7.5‰ group. At the 12 h sampling time, significant differences were only observed between 7.5 and 15‰ groups. The 24 and 96 h sampling time only showed significantly higher differences in the 15‰ group compared to the control for CAT activities (*p* < 0.05).

MDA exhibited significant differences at the beginning of sampling (0 h) between all the groups (*p* < 0.05). From the 4 h to the 96 h sampling time, significantly lower differences were observed in the 15‰ group compared to the control (*p* < 0.05). TAOC activities displayed significant variations between all the treatment groups at the beginning of sampling (0 h) (*p* < 0.05). At the 12 and 24 h sampling times, no differences were observed between the 7.5‰ and 15‰ groups; however, their activities were significantly lower compared to the control (*p* < 0.05). The 48 h and 96 h sampling times had a similar trend of displaying significant differences between all the groups. However, their activities in the 7.5 and 15‰ groups were significantly lower compared to the control with the 7.5‰ group having the lowest expression (*p* < 0.05).

Na+/K+-ATPase activities showed significant differences between all the groups at the beginning of sampling (0 h) (*p* < 0.05). From the 12 h to the 48 h sampling time, the lowest significant differences were observed in the 15‰ group compared to the control (*p* < 0.05). However, no differences were observed between the 7.5 and 15‰ groups.

### 3.4. Digestive Lipase Activities, Amylase, and Pepsin

**Figure 5:**
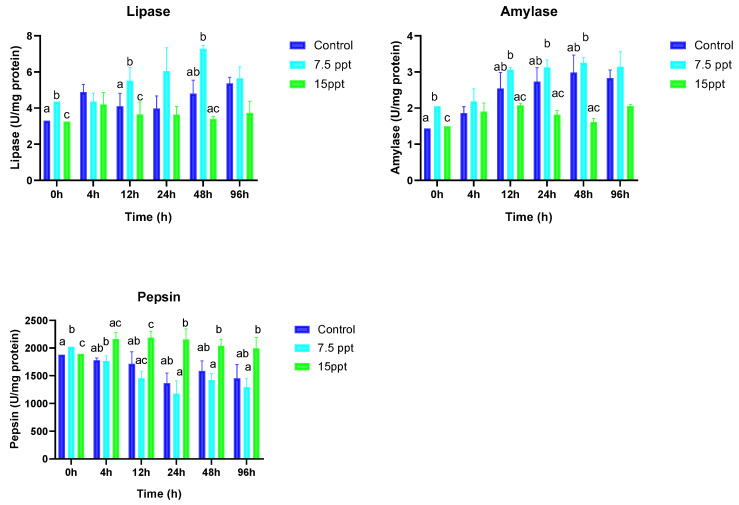
Mean activities of digestive lipase, amylase, and pepsin in *A. grunniens* under varying salinity conditions across different time points. Data are presented as means and standard errors (SEs). Different lowercase letters indicate statistically significant differences between the salinity treatments (*p* < 0.05), while matching letters indicate no significant difference according to two-way ANOVA. A combination of letters (e.g., ‘ab’) indicates that the group shares statistical similarities with both the ‘a’ and ‘b’ groups.

The digestive enzyme activities were substantially influenced by the salinity and time of sampling as shown in Figure 5. In the study, digestive lipase activities displayed significantly higher differences in the 7.5‰ group at the 0 h and 12 h sampling times (*p* < 0.05) compared to the control. At the 0 and 12 h sampling time, the lowest significant differences were observed in the 15‰ group. Amylase activities at the beginning of sampling time exhibited significant differences between all the groups (*p* < 0.05). From the 4 h to the 96 h sampling time, amylase activities followed a similar trend of showing no significant differences between the 7.5‰ and 15‰ groups. Pepsin activities displayed significant differences between all the groups at the beginning of sampling (0-h) (*p* < 0.05). At the 4 h sampling time, significant variations were observed only between the 7.5‰ and 15‰ groups (*p* < 0.05). Pepsin activities at the 12 h sampling time showed significantly higher differences in the 15‰ group compared to the control (*p* < 0.05).

### 3.5. Gill and Liver HSP 70 and HSP 90

**Figure 6:**
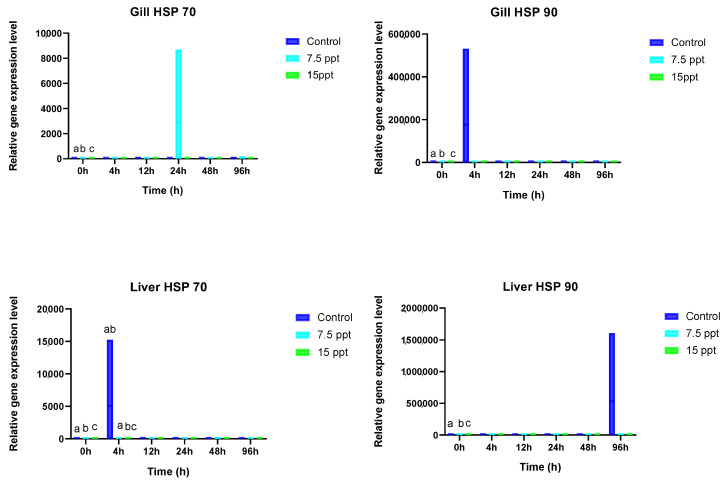
Mean activities of gill and liver heat shock proteins (HSP) 70 and (HSP) 90 in *A. grunniens* under varying salinity conditions across different time points. Data are presented as means and standard errors (SEs). Different lowercase letters indicate statistically significant differences between the salinity treatments (*p* < 0.05), while matching letters indicate no significant difference according to two-way ANOVA. A combination of letters (e.g., ‘ab’) indicates that the group shares statistical similarities with both the ‘a’ and ‘b’ groups.

The HSP 70 and 90 activities in the gills and livers of *A. grunniens* were substantially impacted by salinity and time as displayed in Figure 6. In the gills, HSP 70 and HSP 90 activities followed a similar trend. They exhibited significant variations only at the beginning of sampling (0 h) between all the groups (*p* < 0.05), with no noticeable distinctions during the other sampling times. The liver HSP 70, on the other hand, displayed significant differences at the beginning of sampling (0 h) between all the groups (*p* < 0.05). From the 4 h to the 96 h sampling time, no significant variations were in the salinity treatment groups.

## 4. Discussion

Salinity change is a key eco-stressor for aquatic species, yet some may endure salinity fluctuations depending on how they survive. The current study investigates the effects of varying salinities on *A. grunniens*, the only species in its family that can only survive in freshwater, by assessing the biochemical, antioxidant, and digestive enzyme activities as well as the heat shock protein genes (HSP 70 and 90) in the blood, liver, gills, and intestines. Most physiological functions and biological processes are disrupted by salinity challenges, including hepatic, renal, and gill functions; blood dissolved gases; blood parameters; and hormone control [13]. Furthermore, tolerance to salinity stress varies depending on the aquatic species.

From the observations under the nine different salinity levels, it was shown for the first time that *A. grunniens* can tolerate a range of salinity up to 15‰. An increase in salinity above 15‰ led to a significant mortality rate among *A. grunniens*. This dose-dependent mortality demonstrates *A. grunniens’s* partial tolerance to salt beyond 15‰. Behaviors such as hyperactivity, jumping, and erratic swimming were observed beyond 15‰, indicating that the fish were nearing their tolerance threshold [45]. Increased opercula movement, a sign of respiratory distress, was noted from the 17.5‰ group. This decreased opercular frequency prompted the fish to swim closer to the water surface to enhance oxygen intake [46,47] and consequently caused death within 24 h. Fish hyperactivity in response to chemical stress, particularly in the initial stages, is a common occurrence [48]

Although Kefford et al. [49] noted that the fish mortalities observed in laboratory conditions may not accurately reflect survival capacities in natural environments or represent the maximum salinity levels tolerable by a population, particularly under abrupt salinity changes, the present experiment demonstrated that the different salinity levels tested provide useful insights into the salinity tolerance range of *A. grunniens*. A longer salinity experiment may be required to observe the physiological response of *A. grunniens*. On the other hand, fish exhibited no significant signs of stress up to a salinity of 15‰, suggesting physiological resilience within this range. According to reports, *Catla catla* and *Labeo rohita* fry and fingerlings showed tolerance to 8‰ salinity without any fatalities; however, their survival rates gradually declined as the salinity levels increased [50]. From inference, the death of juvenile *A. grunniens* above 15‰ in this experiment can be associated with excessive exposure to high salinity levels beyond the species threshold compared to when kept in tolerable environments [51,52]. The decline in survival rates as salinity levels increase is a typical trait observed in freshwater stenohaline fish, with the increase attributed to greater osmotic maintenance demands at higher salinities [53].

Plasma ALT and AST, classified as aminotransferase enzymes, serve as universal indicators of liver function in vertebrates by reflecting their concentrations in the blood [54]. These enzymes also provide insights into the stress response in fish, with increased activities signifying potential stress [54]. In the study, the initial sampling at 0 h showed a gradual rise in the ALT and AST levels, with the most significant increase occurring at 15‰. This is expected, as sudden salinity changes can elevate stress enzyme activity. This suggests that the fish may have experienced physiological disturbances related to the need for amino acid compensation and synthesis. Numerous studies have demonstrated that exposure to more saline environments increases AST and ALT activity in common carp (*Cyprinus carpio*), with these enzyme levels rising progressively in response to salinity increases of 5, 10, and 15‰ [55]. Similar patterns were observed in goldfish (*Carassius carassius*), where AST and ALT activities increased as adults transitioned from 0.1 g/L to higher salinities of 4, 8, and 12 g/L [56]. Roche et al. [57] also reported elevated transaminase activities in seabass (*Dicentrarchus labrax*) acclimated to 5 g/L salinity. Likewise, Sultan [58] found that plasma ALT and AST levels rose in the juveniles of *Acanthopagrus latus* reared in salt solutions of 3, 23, and 30 g/L. The lowest significant difference at 15‰ may indicate that this level is near the upper tolerance limit for the species, which contrasts with findings from other studies [56]. This might also suggest that high salinity concentrations could inhibit harmful pathogens that could cause physiological disorders, or that the salt concentration approaches an isoosmotic level needed by the fish. Nonetheless, fish are capable of maintaining homeostasis, preventing severe cellular damage or mortality while keeping ALT levels low, as long as salinity increases stay within acceptable limits [59]. AST activity at 7.5‰ showed significantly higher differences at the 96 h sampling point, indicating a need for further long-term research to examine these trends thoroughly.

In aquatic organisms, variations in salinity have been investigated as a factor that can trigger oxidative stress, characterized by an imbalance between prooxidants and antioxidants [60]. This, in turn, may result in oxidative damage in many instances. The reactive species scavengers SOD and CAT play a crucial role in protecting cells from damage [61]. Maintaining a dynamic balance between normal metabolism and the production and elimination of ROS is essential. Antioxidant enzymes, such as SOD and CAT, effectively eliminate excessive ROS, thereby mitigating the harm caused by lipid peroxidation [62]. In this study, the highest significant differences observed in the SOD activities at the 0 h sampling time were seen in the 15‰ group followed by the 7.5‰ group when compared to the control. This may be indicative of the fact that oxidative stress was activated or an indicator of surplus ROS [4,63]; however, this stress may be higher in the 15‰ group. As time proceeded, no differences were found between the control and the treatment groups, which may mean that salinity may induce higher oxidative stress, but the SOD activities were promptly activated to prevent cell damage. This phenomenon is following the results of a previous study [64]. SOD activities at 96 h displayed a lower significant difference in the 7.5 and 15‰ groups compared to the control. The lower significant differences observed in the latter period may indicate that after being activated, the enzyme level may return to its lower level if the stress is too low to cause any effect or if they are within the tolerable ranges of the fish [65].

Significant differences were observed in CAT activities at the beginning of the sampling with the lowest activities observed in the 7.5‰ group followed by the 15‰ group when compared to the control. This lower significant activity may suggest that *A. grunniens* did not experience elevated stress levels. In *Anoplopoma fimbria,* the antioxidant ability declined with increasing salinity [60]. This observation aligns with Martinez-Alavarez et al. [66], who reported a marked decrease in CAT activities in the liver of the Adriatic sturgeon (*Acipenser naccarii*) as salinity levels increased. After 4, 12, and 96 h sampling times, the CAT activities increased significantly in the 15‰ group compared to the control and the 7.5‰ group. The simultaneous elevation in CAT activity within the 15‰ group, irrespective of time, could be ascribed to the stress conditions induced by heightened free radicals from increased salt concentration. This assertion is in line with various investigations [67,68,69], suggesting a correlation between the alterations in CAT levels and the presence of free radicals inducing stress in fish, and vice versa [70]. Tian et al. [71] noted a decline in CAT activity in the liver with rising salinity levels beyond 6‰, although SOD activity and lipid peroxidation in the liver remained unaffected by water salinity in yellow drum. These observations suggest that the oxidative stress response of fish to changes in salinity is species-specific and can be influenced by factors such as water salinity, developmental stage, experimental conditions, and the adaptation period of fish.

MDA is the end decomposition product of lipid peroxidation. High MDA levels in biological systems can damage cells by binding them to and clumping together proteins and nucleic acids. Therefore, MDA levels indicate the extent of cellular damage caused by free radicals [72] and serve as a crucial indicator of oxidative stress and antioxidant status in cells [73,74]. In the current study, as the salinity levels increased, the MDA levels initially increased at the 0 h mark, likely due to baseline metabolic activities or minor stressors, before decreasing. This indicates that while lipid peroxidation from rising salinity can elevate MDA degradation, the initial increase in the control may stem from processes unrelated to salinity stress. Further research is needed to clarify these dynamics and their implications for the freshwater drum’s environmental responses. Nevertheless, regardless of the length of time, the MDA levels in the 7.5 and 15‰ groups remained low compared to the control starting at the 4 h sampling interval. This lower level may indicate a balance between producing and removing ROS or very little damage from the stress as this phenomenon agrees with [75]. Alternatively, the diminished MDA levels may indicate a reduced rate of lipid peroxidation at higher salinity levels or a lack of stress-induced damage in fish cells [74,76]. The reduction in MDA levels under varying salinity conditions aligns with previous studies by Chen et al. [77].

The TAOC is associated with modulating the activities of SOD and CAT activities during various developmental stages [78]. It functions as a pivotal gauge for evaluating the comprehensive antioxidant capability of the body, with its extent reflecting the body’s capacity to counteract free radical metabolism brought about by external stimuli [79,80]. In the study, notably higher TAOC activities were observed at the onset of sampling (0 h) in the treatment groups compared to the control. This phenomenon serves as a compensatory response to the stress induced by osmotic changes, primarily due to the activation of antioxidant enzymes such as SOD and CAT, which help mitigate oxidative damage caused by ROS generated under stress conditions [18,81]. The decreased significant activities observed in the 7.5‰ and 15‰ groups compared to the control starting from the 4 h sampling time may suggest that changes in salinity levels could have induced stress in the fish. However, these circumstances did not result in extensive harm to the fish. The antioxidant capacity of *Anoplopoma fimbria* diminishes with rising salinity [60]. Long-term studies on the effects of salinities on the antioxidant capacities of *A. grunniens* is required.

The Na+/K+-ATPase is a vital membrane protein in aquatic organisms, regulating ion balance and overall osmoregulation. Its activity is significantly affected by salinity [82,83]. This pump, primarily composed of Na+/K+-ATPase enzymes, actively transports Na+ and K+ across cell membranes. Changes in Na+/K+-ATPase enzyme activity and osmotic pressure are the key indicators of a fish’s salinity adaptation. While Na+/K+-ATPase typically facilitates Na+ and Cl- uptake in freshwater environments, it may play a role in excreting excess Na+ and Cl- in high-salinity conditions [84]. The increased activities observed at the commencement of sampling in the 7.5‰ and 15‰ compared to the control may be attributed to metabolic shifts, initiating Na+/K+-ATPase activation until osmotic pressure reaches equilibrium. Hypersalinity triggers changes in the osmoregulatory processes of the gills by activating the Na+/K+-ATPase pump. This response is viewed as a compensatory mechanism aimed at enhancing ion absorption. Thus, the increased activity of Na+/K+-ATPase in the gills is essential for effective acclimatization to higher salinity levels [85]. As a result, there is an enhancement in energy production by ATPase synthase, which is crucial for ATP generation. This high level of energy production acts as a primary trigger for the Na+/K+-ATPase pump, facilitating ion exchange during hypersalinity and ultimately leading to tolerance [86].

Additionally, increased environmental salinity alters the ultrastructure of chloride cells, which play a vital role in acid–base regulation under high salinity by managing Cl-/HCO3- exchange. Previous research has demonstrated that exposure to salinity boosts both the number and activity of chloride cells in the gills of species such as common carp [55], *Oreochromis mossambicus* [87], and juvenile Australian snapper [88]. The significantly lower Na+/K+-ATPase activities noted in the 7.5 and 15‰ at the 24 and 48 h sampling time compared to the control imply a decrease in the gradient between body fluid and the environment over time, resulting in reduced energy for osmoregulatory activities. This finding corresponds with the outcomes reported on Atlantic sturgeon [66]. While Na+/K+-ATPase primarily participates in Na+ and Cl− uptake in freshwater, it may also be involved in the secretion of excess Na+ and Cl− in hypertonic water [83].

In fish, digestive enzymes produced by the organs of the digestive system play a crucial role in facilitating nutrient absorption and conversion, thereby enhancing digestion. Salinity can influence the specific activities of these enzymes. Specifically, changes in salinity alter the osmotic pressure within a fish’s body, which impacts the activity of gastrointestinal digestive enzymes [89,90]. Gheisvandi et al. [91] found that salinity can significantly affect both the specific activities and efficiency of these digestive enzymes. Their responses vary across species, life stages, and exposure duration, but generally, low salinities inhibit the protease, amylase, and lipase function, hampering digestion and growth [75,92,93]. However, some euryhaline species show increased activity at lower salinities, demonstrating adaptation capacity [94]. Broadly, salinity fluctuations within tolerance limits prompt three enzyme response types: activation, inhibition, or no change [95,96]. Higher lipase activities were noted between the 0 and 12 h sampling points, with the highest activity observed in the 7.5‰ salinity treatment, followed by the 15‰ treatment, compared to the control group, but no differences were observed in the other sampling times. In certain species like *A. latus* and *L. calcarifer*, lipase activities have been reported to increase with rising salinity levels up to specific thresholds of 35 and 24 salinities, respectively, before declining at higher salinities [97]. Although limited studies have explored the effects of salinity on lipase, initial findings suggest that enzyme responses may vary considerably across species [98]. Further research on *A. grunniens* is warranted, ideally incorporating feeding, to elucidate the long-term effects of salinity.

Amylase activities showed a higher significant difference at the beginning of sampling (0 h) with the highest observed in the 7.5‰ group followed by the 15‰ group. The higher amylase activities observed at the beginning of sampling may reflect an initial physiological response aimed at coping with the osmotic challenges posed by the higher salinity levels, or the possibility of fish mobilizing resources to facilitate the digestion and absorption of carbohydrates, which can provide the energy needed for osmoregulation and other physiological processes. The no differences observed between the 7.5‰ group and the control across the different sampling times indicate that amylase retained its ability to catalyze the breakdown of starch into simpler sugars at the 7.5‰ level regardless of time. Salinity showed no apparent effect on the unique activity of amylase in any of the five salinity groups of juvenile American shad *Alosa sapienssima* [99]. Though patterns may differ between species, reduced amylase function under high salinities or chloride concentrations is commonly documented [100,101].

Pepsin activities on the other hand at the beginning of the sampling period (0 hours) significantly increased in the 7.5 and 15‰ groups compared to the control. This could reflect the abrupt mobilization of resources to changes in salinities. Unchanged pepsin activity at 7.5‰ over time indicates this level did not inhibit function, potentially reflecting internal salt tolerance or near isoosmotic conditions for *A. grunniens*. Increased pepsin activity at 15‰ may stem from protein catabolism to meet osmoregulatory demands, aligning with other evidence of protein resource utilization under salt stress [52,102]. Previous research in *Sparus aurata* similarly found no impact on pepsin at lower salinities [103]. In summary, *A. grunniens* demonstrates resilience by temporarily activating or retaining digestive capacity over salinity fluctuations within tolerable limits. This helps inform suitable salinity ranges for culture and release.

HSPs are recognized as potential biomarkers for environmental stress in fish [24,104]. Proteins like HSP 70 and HSP 90 are typically present at low levels in unstressed cells, and their expression generally increases following exposure to stressors [105,106]. When cells encounter various stressors such as toxic metal contamination, hypoxia/anoxia, chemical shocks, salinity stress, thermal stress, or transportation stress, the rapid upregulation of HSPs enhances cell viability by mitigating or minimizing the harmful effects of these stressors [24,104]. In this study, significant differences were noted in the activities of Hsp 70 and Hsp 90 in the gills of *A. grunniens* at the initiation of the sampling time (0 h), but no discernible changes were observed in their activities as time progressed. These initial differences in activity could be attributed to the roles of these genes in mitigating protein disruption and injury in response to stress. Similar observations have been documented in studies on the silver sea bream, *Sparus sarba* [106], and the brown trout, *Salmo trutta* [107], which detected increased levels of HSP 70 in response to higher osmotic stress. The lack of significant differences in HSP 70 and HSP 90 activities over time regardless of the salinity levels may indicate a threshold effect, where certain conditions are required to induce the overexpression or upregulation of these genes. A study on *Sarotherodon melanotherons* found no significant differences in HSP enzyme levels at salinities approaching 40 psu [108]. Typically, HSP 70 and HSP 90 act as guardians of cellular homeostasis in response to environmental threats, closely linked to stress and salinity conditions [109]. Although no variations were observed over time, numerical fluctuations in enzyme activity may suggest ongoing protein turnover. However, the salinity levels experienced by *A. grunniens* may be within tolerable limits, potentially insufficient to trigger significant increases in the HSP 70 or HSP 90 levels. The sharp increase observed in the gill HSP 90 activity after 4 h may be attributed to the gene’s inherent inducibility characteristics in cellular protection in response to environmental fluctuations [110]. Finally, the absence of differences between the salinity levels of 7.5‰ and 15‰ may suggest that the genes have adapted to the environment over time.

There were notable variations in the liver activities of HSP 70 and HSP 90 at the initial sampling time (0 h) across all the groups. However, no significant differences were observed over time, irrespective of the salinity levels. These initial variations may be attributed to individual differences in metabolic responses or stress levels in the fish at the start of the experiment, which could influence the expression of heat shock proteins. They may also potentially indicate the protective role of HSPs against liver cell damage and inflammation. This observation aligns with the findings by Ramaglia and Buck [111] regarding the protective effects of HSPs against inflammation damage in reptiles. The notable increase in the liver HSP90 activity may be directly linked to the metabolism of stress proteins, suggesting a redirection of energy from growth towards osmoregulation by stress proteins, a phenomenon observed in many cases. This metabolic effect on stress protein activity and its relation to increased osmoregulatory activities is supported by the studies conducted by Basu et al. [25,104]. The absence of significant differences observed may indicate the adaptive nature of HSP genes in different environmental conditions, particularly in response to constant salinities. This adaptability of HSPs to diverse environmental conditions is corroborated by reports from Padmini and Rani, [112]. However, further studies over extended periods are warranted as HSP levels tend to increase over time [113]. *Potamocorbula amurensis* exposed to 6.5‰ salinity, HSP 70–72 levels were not significantly different from those in clams treated with 0.5 or 25‰ salinity, although the average hsp70–72 levels were similar to those in clams exposed to 25‰ in [114]. The absence of significant differences across all the sampling points could also suggest that regardless of salinity and time, the fish were not severely stressed or that the stress levels were insufficient to cause significant changes in liver HSP activities in the treatment groups compared to the control.

## 5. Conclusions

This experiment provides important new data on the physiological responses of the freshwater drum to varying salinity levels. Understanding the tolerable ranges for this species is crucial for effectively addressing the related health challenges and expanding its aquaculture potential. The initial observations of mortality in *A. grunniens* indicate its tolerance to salinity levels of up to 15‰ without experiencing any physical harm. Furthermore, the study emphasizes the species’ ability to manage oxidative and peroxidative stressors within the salinity range of 7.5 to 15‰, as shown by its biochemical ALT and AST activities when compared to freshwater habitats. Additionally, the varying salinity levels resulted in lower MDA activities. Antioxidant activities fluctuated across different salinity levels, but they effectively countered the stress induced by these variations. Our findings on antioxidant responses are consistent with those reported by Chen et al. [77], who observed a significant increase in antioxidant enzyme activities under varying salinities, suggesting a response to oxidative stress. *A. grunniens* also demonstrates adaptability in its digestive capacity at salinity levels of 7.5 and 15‰. The expressions of HSP 70 and 90 in the gills and livers further indicate that *A. grunniens* can initiate appropriate physiological responses when faced with environmental stresses such as changes in salinity. As the only species in its family permanently adapted to freshwater conditions, the overall health of *A. grunniens* observed across various salinity levels over extended periods suggests its ability to thrive in diverse conditions, including brackish water, up to 15‰. Further research into growth and physiological responses should be conducted over extended durations, including feeding trials, to further assess the adaptability of *A. grunniens* to varying salinities.

## Figures and Tables

**Table 1:** Primers used for qRT-PCR verification of differently expressed genes.

Gene	Primers
β-actin	F:5′-AGGCTGTGCTGTCCCTGTAT-3′
	R:5′-GCTGTGGTGGTGAAGGAGTAC-3′
HSP 70	F:5′-ATTGCTGAAGCCTACCTCGG-3′
	R:5′-GTGCCTCCACCGAGATCAAA-3′
HSP 90	F:5′-CTTTGCCACGTTCTGGAAGC-3′
	R:5′-CCAGACCTCCAACAGCGAAA-3′

Notes: beta-actin (β-actin), HSP 70, and HSP 90 genes forward (F) and reverse (R) primer sequence.

## Data Availability

The authors confirm the availability of data supporting the findings of the current study within the manuscript upon request.
